# Orbital Abscess With Vision Threatening Optic Neuropathy Due to Odontogenic Sinusitis in Adolescence: A Case Report and Literature Review

**DOI:** 10.7759/cureus.71664

**Published:** 2024-10-16

**Authors:** Nur Faizah Harun, Wan-Hazabbah Wan Hitam, Mohd Feendi Mohd Fauzi Yap, Juliana Jalaluddin

**Affiliations:** 1 Department of Ophthalmology and Visual Science, School of Medical Sciences, Universiti Sains Malaysia, Kelantan, MYS; 2 Department of Ophthalmology, Hospital Melaka, Melaka, MYS

**Keywords:** odontogenic infection, optic neuropathy, orbital abscess, pediatrics, streptococcus intermedius

## Abstract

Orbital abscesses secondary to odontogenic infections are rare but can lead to serious complications, including compressive optic neuropathy and permanent vision loss, if not diagnosed and treated promptly. We present the case of a 13-year-old child with a radiologically confirmed orbital abscess associated with a recent odontogenic infection. The patient initially presented with a one-week history of right eyelid swelling and fever. The symptoms began after treatment for dental caries, during which the patient was prescribed a three-day course of oral antibiotics. Despite emergency pulp extirpation, the swelling worsened, leading to severe proptosis, decreased vision, and mild optic nerve dysfunction. Surgical exploration of the right orbit with incision and drainage revealed 8 mL of thick purulent material. Culture results confirmed *Streptococcus intermedius* as the causative organism. Following a combination of intravenous antibiotics and surgical intervention, the patient showed significant improvement, with resolution of swelling and restoration of vision. This case highlights the critical importance of early diagnosis, timely imaging, and surgical and medical management integration in preventing vision-threatening complications of orbital abscesses caused by odontogenic infections in pediatric patients.

## Introduction

An orbital abscess is a localized collection of pus within the orbital space surrounding the eye, often arising from the spread of infections in adjacent structures, particularly those of dental origin (odontogenic infections) and the paranasal sinuses [1]. These infections can extend into the orbit, leading to serious complications if not promptly treated. Orbital abscesses are more commonly seen in adolescents, who exhibit more pronounced and aggressive symptoms than adults [2]. Due to the proximity of the orbital space to critical anatomical structures, early diagnosis and intervention are crucial to preventing severe outcomes, including vision loss or intracranial extension [3].

Orbital abscess, if not promptly diagnosed and treated, can lead to devastating complications, including vision loss, intracranial infections, and even death. Early identification and intervention are critical in improving patient outcomes and preventing life-threatening sequelae. In this case, *Streptococcus intermedius* - a rare pathogen in orbital abscesses - was identified as the causative organism. This unusual finding underscores the potential for *S. intermedius* to cause aggressive, severe infections, highlighting the importance of early recognition and swift intervention to avoid serious complications.

## Case presentation

A previously healthy 13-year-old boy was referred to the ophthalmology team due to a one-week history of right eyelid swelling, accompanied by fever. Despite receiving oral antibiotics for three days, the swelling worsened. The patient had a recent history of treated dental caries one week before the onset of symptoms but denied any recent nasal or ear discharge, insect bites, or ocular trauma. On examination, the patient was comfortable, well-hydrated, and afebrile, with a recorded temperature of 37.9°C.

On initial assessment, the patient’s visual acuity was 6/9 bilaterally. Examination of the right eye revealed significant upper eyelid swelling and erythema, causing mechanical ptosis. The eyelid was warm and tender to palpation, and mild proptosis (2 mm) was observed. Ocular motility was intact with no restriction in movement. The conjunctiva was injected and mildly chemosed, but there was no anterior chamber reaction. Intraocular pressure (IOP) was measured at 18 mmHg in the right eye and 16 mmHg in the left, with normal optic nerve function. Examination of the left eye was unremarkable.

**Figure 1:**
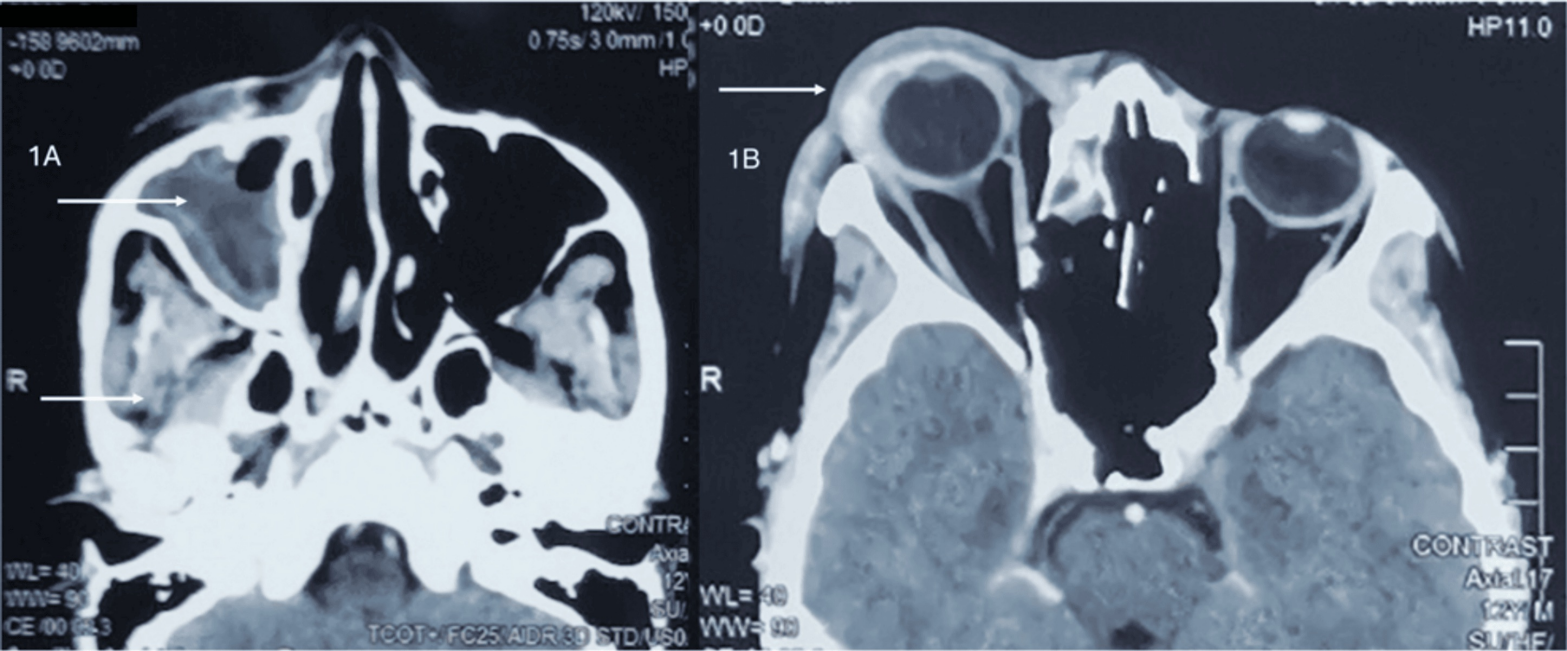
Axial section of the orbital CT scan revealing a large, multiseptated abscess in the right superior extraconal region, opacification of the right maxillary sinus (1A), and proptosis of the right globe (1B).

**Figure 2:**
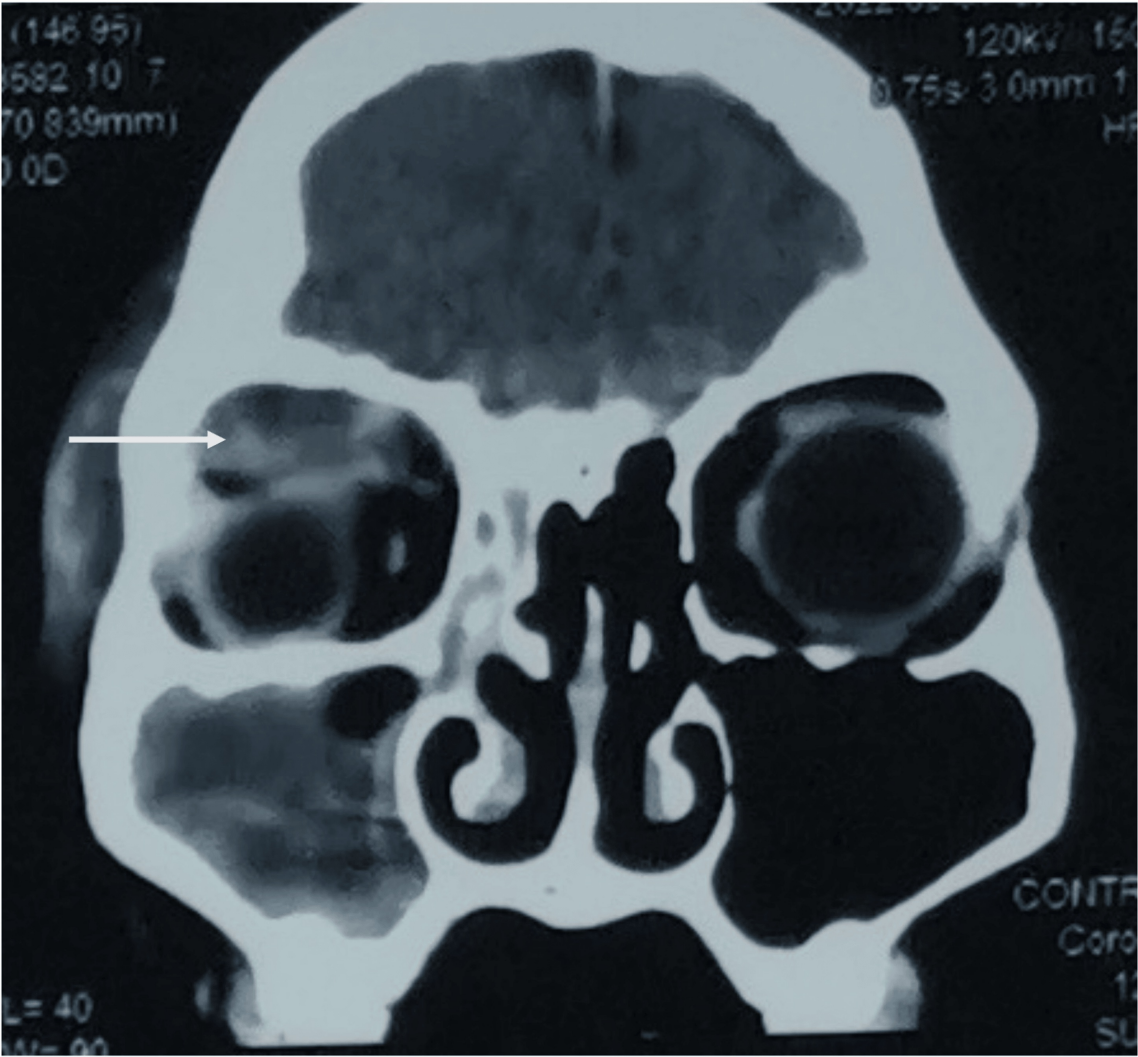
CT scan revealing a large abscess at the superior extraconal region (arrow) causing compression to the right eye globe.

The patient was admitted and started on intravenous amoxicillin/clavulanic acid. Urgent contrast-enhanced computed tomography (CECT) of the brain, orbits, and paranasal sinuses revealed a large multiseptated abscess in the right superior extraconal space, along with right-sided paranasal sinusitis (Figures 1, 2).

A dental evaluation by the oral and maxillofacial surgery team confirmed apical periodontitis in tooth 16, leading to an emergency pulp extirpation. Blood investigations revealed an elevated white blood cell count with neutrophil predominance (Table 1).

**Table 1:** Complete blood count test RBC, red blood cell; WBC, white blood count; MCV, mean corpuscular volume; MCH, mean corpuscular hemoglobin; MCHC, mean corpuscular hemoglobin concentration

Test	Result	Unit	Reference range
Hemoglobin	130	g/L	120–150
RBCs	4.41	10^12^/L	3.80–4.80
Hematocrit	40.4	%	36.0–46.0
MCV	92	fL	83–101
MCH	29.5	pg	27.0–32.0
MCHC	32.2	g/dL	31.5–34.5
Platelets	250	10^9^/L	150–410
WBCs	13.6 (high)	10^9^/L	4.0–10.0
Neutrophils	9.6 (high)	10^3^/uL	2.0–7.0
Lymphocytes	2.6	10^3^/uL	1.0–3.0
Monocytes	0.9	10^3^/uL	0.2–1.0
Eosinophils	0.2	10^3^/uL	0.0–0.5
Basophils	0.07	10^3^/uL	0.01–0.08

Based on clinical and radiological findings, the diagnosis of an orbital abscess was made. The antibiotic regimen was escalated to intravenous ceftazidime (1 gram daily) and intravenous metronidazole (500 mg every eight hours). Topical moxifloxacin 0.5% was administered every six hours for the right eye, and oral paracetamol (1 gram every six hours) was given for pain relief. Regular warm compresses were applied, and daily assessments were conducted to monitor optic nerve function.

**Figure 3:**
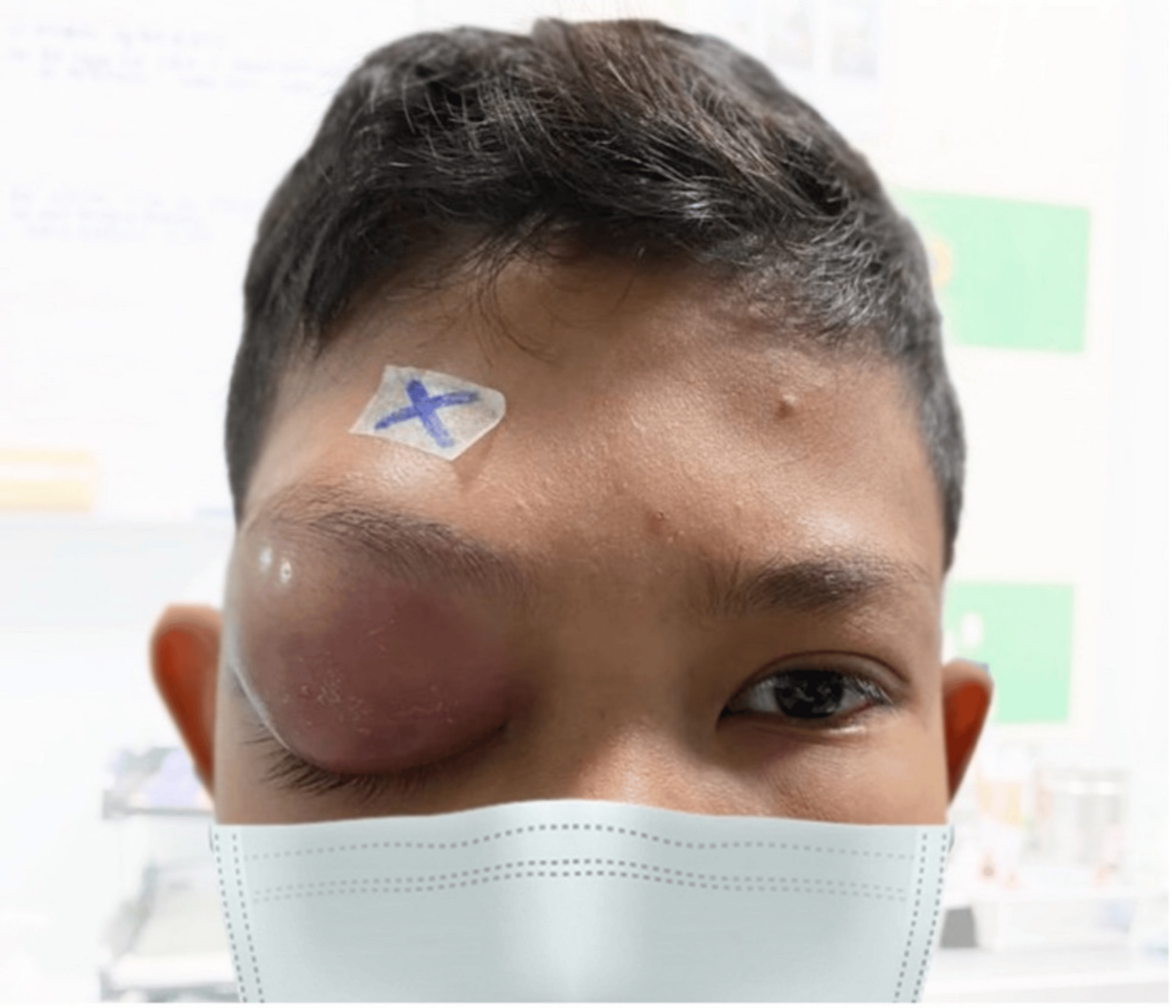
Worsening of right eye swelling with complete ptosis, severe proptosis, and deterioration of optic nerve function.

On the second day of admission, the patient’s right eye visual acuity deteriorated to 6/24, with stable vision at 6/9 in the left eye. A relative afferent pupillary defect (RAPD) was detected in the right eye, along with decreased brightness of light perception and red desaturation (Figure 3). Eye movements became restricted, and IOP in the right eye rose to 25 mmHg. Topical timolol, administered twice daily, was initiated to control IOP.

An emergency wound exploration with incision and drainage of the right orbit was performed, yielding 8 mL of thick pus. Culture results identified *S. intermedius* (Gram-positive cocci) as the causative organism, which was sensitive to chloramphenicol, erythromycin, and penicillin G antibiotics. Following surgery, the patient showed improvement in both visual acuity and optic nerve function in the right eye.

**Figure 4:**
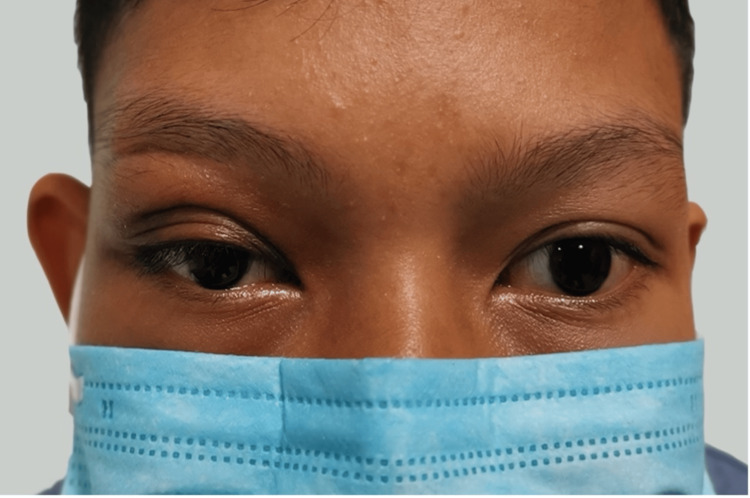
Post-operative image at two weeks.

The patient completed a one-week course of intravenous antibiotics and was discharged on an additional one-week course of oral cefuroxime 250 mg twice daily. At follow-up, the patient’s condition had significantly improved, with restored vision and no further complications (Figure 4).

## Discussion

Orbital abscesses can arise from various sources, including odontogenic, periorbital, sinonasal, or systemic infections. If left untreated, these infections may lead to severe, potentially life-threatening complications such as cavernous sinus thrombosis, cerebral abscess, and permanent vision loss [4,5]. In the present case, the infection originated from apical periodontitis and paranasal sinusitis. Timely identification of these sources is crucial to preventing complications and ensuring favorable outcomes.

In this case, *S. intermedius* was identified as the causative organism. This bacterium, although relatively uncommon in orbital abscesses, is part of the *Streptococcus anginosus* group, known to cause abscesses in deep tissues such as the brain and liver. Previous studies have shown that *Streptococcus *species are the most frequently isolated pathogens in orbital abscesses, accounting for up to 60% of cases, followed by *Staphylococcus aureus *and *Pseudomonas aeruginosa *[6,7]. The identification of *S. intermedius* in this case highlights the importance of recognizing rare pathogens and tailoring antibiotic therapy accordingly.

In this patient, the abscess was traced to a combination of apical periodontitis and paranasal sinusitis, which underscores the role of dental infections as a significant contributor to orbital abscesses. The infection likely spread from the dental apex to the orbit through the surrounding bone, emphasizing the need for early dental evaluation in cases of orbital infection with a suspected odontogenic origin [8]. Timely dental intervention, including the emergency pulp extirpation performed in this case, is critical to halting the spread of infection.

Imaging plays a pivotal role in diagnosing and managing orbital abscesses, as clinical signs alone may be insufficient to determine the extent and severity of the infection. CECT is the imaging modality of choice, providing detailed information about the location, size, and extent of the abscess [5]. In this case, CECT revealed a large multiseptated abscess in the right superior extraconal space, as well as right-sided paranasal sinusitis, without intracranial or cavernous sinus involvement. This imaging result allowed for targeted treatment, helping avoid more severe complications such as vision loss or intracranial extension. Studies show that up to 10% of patients with orbital abscesses may experience blindness if treatment is delayed, underscoring the importance of early diagnosis and prompt intervention [6].

Despite initial intravenous antibiotic therapy, the patient's condition deteriorated, with worsening visual acuity, development of RAPD, and increased IOP. This clinical decline reflected the need for more aggressive management, as it suggested the onset of compressive optic neuropathy, a potential precursor to irreversible vision loss. The close monitoring of optic nerve function was essential in recognizing the need for surgical intervention at the appropriate time.

Surgical drainage is often necessary in the management of orbital abscesses, particularly when medical therapy alone is insufficient. Studies, including those by Sciarretta et al. and Zawadzki et al., emphasize that surgery is required in most cases, with the choice of technique determined by the abscess’s location [4,5]. In this case, the patient did not respond adequately to intravenous antibiotics, necessitating an incision and drainage procedure through the right upper eyelid to relieve pressure and remove the accumulated pus. This timely surgical intervention prevented further complications and led to significant clinical improvement.

If not treated promptly, orbital abscesses can lead to devastating complications such as cavernous sinus thrombosis, cerebral abscess, or permanent vision loss. Fortunately, in this case, a multidisciplinary approach involving both medical and surgical treatment resulted in a successful outcome. The patient completed a course of intravenous and oral antibiotics following the incision and drainage procedure, leading to full recovery of visual acuity and resolution of the infection.

This case highlights the importance of early recognition and intervention in managing orbital abscesses, particularly those arising from dental infections. The combination of appropriate imaging, prompt antibiotic therapy, and surgical drainage is crucial to preventing severe complications. Moreover, the identification of the causative pathogen, *S. intermedius*, reinforced the need for microbiological analysis to guide therapy. A multidisciplinary approach, including ophthalmology, maxillofacial surgery, and radiology, was instrumental in achieving a successful outcome for the patient.

## Conclusions

This case highlights the significant impact that early intervention, including timely imaging, accurate pathogen identification, and appropriate surgical management, can have on patient outcomes in orbital abscesses. Rapid diagnosis and treatment are essential for preventing potentially life-threatening complications. A multidisciplinary approach involving dental, ophthalmological, radiological, and surgical expertise was instrumental in achieving a successful outcome. Prompt intervention not only resolved the infection but also preserved vision, emphasizing the need for coordinated care to mitigate long-term risks such as permanent vision loss.
